# Cationic Amino Acid Transporter 2 Enhances Innate Immunity during *Helicobacter pylori* Infection

**DOI:** 10.1371/journal.pone.0029046

**Published:** 2011-12-14

**Authors:** Daniel P. Barry, Mohammad Asim, Brooks P. Scull, M. Blanca Piazuelo, Thibaut de Sablet, Nuruddeen D. Lewis, Lori A. Coburn, Kshipra Singh, Lesley G. Ellies, Alain P. Gobert, Rupesh Chaturvedi, Keith T. Wilson

**Affiliations:** 1 Division of Gastroenterology, Department of Medicine, Vanderbilt University Medical Center, Nashville, Tennessee, United States of America; 2 Veterans Affairs Tennessee Valley Healthcare System, Nashville, Tennessee, United States of America; 3 Department of Cancer Biology, Vanderbilt University Medical Center, Nashville, Tennessee, United States of America; 4 Department of Pathology, University of California San Diego, La Jolla, California, United States of America; 5 Institut National de la Recherche Agronomique, Unité de Microbiologie UR454, Saint-Genès-Champanelle, France; 6 Department of Pathology, Microbiology and Immunology, Vanderbilt University Medical Center, Nashville, Tennessee, United States of America; Indian Institute of Science, India

## Abstract

Once acquired, *Helicobacter pylori* infection is lifelong due to an inadequate innate and adaptive immune response. Our previous studies indicate that interactions among the various pathways of arginine metabolism in the host are critical determinants of outcomes following infection. Cationic amino acid transporter 2 (CAT2) is essential for transport of l-arginine (L-Arg) into monocytic immune cells during *H. pylori* infection. Once within the cell, this amino acid is utilized by opposing pathways that lead to elaboration of either bactericidal nitric oxide (NO) produced from inducible NO synthase (iNOS), or hydrogen peroxide, which causes macrophage apoptosis, via arginase and the polyamine pathway. Because of its central role in controlling L-Arg availability in macrophages, we investigated the importance of CAT2 *in vivo* during *H. pylori* infection. CAT2^−/−^ mice infected for 4 months exhibited decreased gastritis and increased levels of colonization compared to wild type mice. We observed suppression of gastric macrophage levels, macrophage expression of iNOS, dendritic cell activation, and expression of granulocyte-colony stimulating factor in CAT2^−/−^ mice suggesting that CAT2 is involved in enhancing the innate immune response. In addition, cytokine expression in CAT2^−/−^ mice was altered from an antimicrobial Th1 response to a Th2 response, indicating that the transporter has downstream effects on adaptive immunity as well. These findings demonstrate that CAT2 is an important regulator of the immune response during *H. pylori* infection.

## Introduction

The Gram-negative bacterium *Helicobacter pylori* is one of the most successful bacterial pathogens of humans. It infects more than 50% of the world's population, and although *H. pylori* infection elicits an immune response, it is insufficient to eliminate the bacteria. Left untreated, infection generally persists for the lifetime of the host and can cause a collection of disorders that arise from the resultant chronic inflammation, including dyspepsia, gastritis, and gastric and duodenal ulcers [1]. It is also strongly associated with development of gastric cancer, the second most common cause of cancer death worldwide [2].

Macrophages are an important component of the innate immune response to *H. pylori* infection [3]. We have demonstrated that cationic amino acid transporter 2 (CAT2) expression is upregulated in macrophages in vitro, and in mouse and human gastritis tissues, with co-localization to gastric macrophages by immunostaining; we also identified CAT2 as the protein responsible for the uptake of l-arginine (L-Arg) into macrophages [4]. We previously reported that within macrophages, L-Arg is the common substrate utilized in two major divergent pathways. Nitric oxide (NO), a potent anti-microbial and signaling molecule, is generated from L-Arg by inducible NO synthase (iNOS) [5] and our previous studies suggest that the host's failure to clear infection is due, at least in part, to insufficient production of iNOS and NO by macrophages [6]. Competing with this enzyme, arginase catalyzes the conversion of L-Arg into l-ornithine, which is subsequently converted to the polyamine putrescine by ornithine decarboxylase (ODC), followed by conversion to the polyamines spermidine and spermine [7]. We also previously found that mice lacking arginase 2 (Arg2) exhibit increased gastritis and lower colonization than wild type mice following infection with *H. pylori* [6].

The innate immune system serves several important functions, including uptake and removal of bacteria and elaboration of cytokines that modulate the adaptive response and affect lymphocyte function. During the response to *H. pylori*, dendritic cells may also be involved as links between the innate and adaptive immune responses [8], [9], [10]. The T cell stimulatory cytokine IL-12p70, composed of a heavy chain (p40) and a lighter chain (p35), is produced by both macrophages and dendritic cells in response to bacteria [11]. It has also been reported that monomers and homodimers of p40 may act as antagonists toward the heterodimer [12]. IL-12p70 is known to promote the Th1 response—characterized by production of IL-2, interferon-γ (IFN-γ—, and tumor necrosis factor-α—and lessen Th2 responses, in which production of IL-4 and IL-13 is induced [13]. In vaccination studies, a strong Th1 response has been shown to be necessary for clearance of *H. pylori* [3], [14].

Based on our earlier findings, we hypothesized that mice lacking CAT2 would exhibit a defective immune response during infection. We now report that CAT2^−/−^ mice chronically infected with *H. pylori* exhibit decreased levels of gastritis and increased bacterial colonization compared to wild type mice. These changes are accompanied by reduced levels of macrophages, iNOS, and activated dendritic cells. We also found that CAT2^−/−^ mice demonstrate impairment in their production of IL-12p70, a cytokine known to stimulate a Th1-type lymphocytic response and suppress Th2 activities, and found evidence for such an effect manifested as changes in expression patterns of other cytokines associated with specific T cell pathways and neutrophil proliferation. These data suggest that a function of CAT2 is to enhance the immune response to *H. pylori* and augment its ability to reduce bacterial load.

## Materials and Methods

### Ethics Statement

This study was carried out following recommendations in the Guide for the Care and Use of Laboratory Animals of the National Institutes of Health. The protocol was approved by the Institutional Animal Care and Use Committee of Vanderbilt University (Protocol number V/07/247).

### Bacterial cultures and mouse infection

*H. pylori* SS1 [15] was maintained by passage on plates of tryptic soy agar containing 5% sheep blood, as described [16]. Male wild-type C57BL/6 (Jackson Laboratories) or congenic CAT2^−/−^ mice [17] aged 6–8 weeks were infected as described [18], [19]. Briefly, *H. pylori* were grown overnight in Brucella broth supplemented with 10% FBS at 37°C with 5% CO_2_ at 120 rpm. For chronic 4-month infections, 5×10^8^ bacteria were delivered intragastrically on days 0, 2 and 4. For acute 48-h infections, a single dose was used. At the pre-defined endpoint, mice were sacrificed, their stomachs were removed, and the glandular portion was divided for downstream analyses.

### Histology

Portions of the stomach were fixed in 10% buffered formaldehyde and embedded in paraffin. Sections were cut, stained with hematoxylin and eosin, and examined microscopically by a blinded pathologist (MBP) to grade histological gastritis. For quantification, chronic (lymphocytic) and acute (granulocytic) gastritis were each measured on a scale of 0–3 in both the gastric antrum and body with the scores added together for a total of 0–12 [4], [20].

### *H. pylori* colonization

Bacterial load in the stomach was determined as described [21]. A weighed portion of the body was disintegrated using a Ultra-Turrax rotor-stator homogenizer. Homogenates were serially diluted and spread on tryptic soy agar plates supplemented with 5% sheep blood, 10 µg/mL naladixic acid, 100 µg/mL vancomycin, 2 µg/mL amphotericin, 200 µg/mL bacitracin, and 2.5 U/mL polymixin B. Plates were incubated for one week at 37°C with 5% CO_2_ at which time colonies were counted to determine bacterial load as colony-forming units (CFU) per gram.

### Flow cytometry

The complete glandular portions from the stomachs of mice infected for 48 h were digested with collagenase (Sigma) and dispase (Roche) and the resulting cell suspension was analyzed by flow cytometry to identify infiltrating immune cells as described [6]. We detected expression of the macrophage marker F4/80 with a phycoerythrin-tagged antibody, the dendritic cell marker CD11c with an allophycocyanin-Cy7-tagged antibody, and major histocompatibility complex (MHC) II with a Pacific Blue-tagged antibody (BD Biosciences).

### Western blot analysis

Portions of frozen body and antrum were suspended in CelLytic MT Cell Lysis Reagent (Sigma) containing EDTA-free Protease Inhibitor Cocktail Set III, and Phosphatase Inhibitor Set I (EMD Chemicals) and disrupted by three 10-second pulses of sonication at 40 W (Ultrasonic Processor GE 130PB, Hielscher). For each mouse, 80 µg of protein were resolved on a 10% polyacrylamide gel (Bio-Rad) and transferred overnight onto PVDF. After blocking with 5% milk for 2 h, iNOS and β-actin were detected by Western blotting as described [22].

### Immunofluorescence

Paraffin-embedded stomach strips were cut into 5-µm sections and iNOS-expressing F4/80^+^ macrophages or CD11c^+^ dendritic cells were identified by immunofluorescence as described [4], [6]. iNOS was detected with a rabbit polyclonal antibody (1∶100 dilution; BD Biosciences) followed by a goat anti-rabbit secondary antibody conjugated to fluorescein isothiocyanate (FITC) (1∶400 dilution; BD Biosciences). F4/80 was detected with a rat polyclonal antibody (1∶50 dilution; Invitrogen) followed by a rabbit anti-rat secondary antibody conjugated to tetramethyl rhodamine iso-thiocyanate (TRITC) (1∶100 dilution; Sigma). CD11c was detected with a monoclonal hamster antibody conjugated to phycoerythrin (1∶50 dilution; Miltenyi Biotec).

### Luminex multiple cytokine assay

Multiple cytokines were quantified in the protein lysates generated for the Western blots using the MILLIPLEX MAP Mouse Cytokine/Chemokine Magnetic Bead Panel (Millipore) according to the manufacturer's instructions, using a Luminex FLEXMAP 3D instrument.

### Statistical analysis

Data are expressed as means ± standard error. Student's *t* test was used for pairwise comparisons. Data from more than two groups (normalized by log transformation, if necessary) were analyzed by ANOVA followed by the Newman-Keuls post hoc multiple comparisons test.

## Results

### Infected CAT2^−/−^ mice exhibit less gastritis and increased gastric colonization

**Figure 1:**
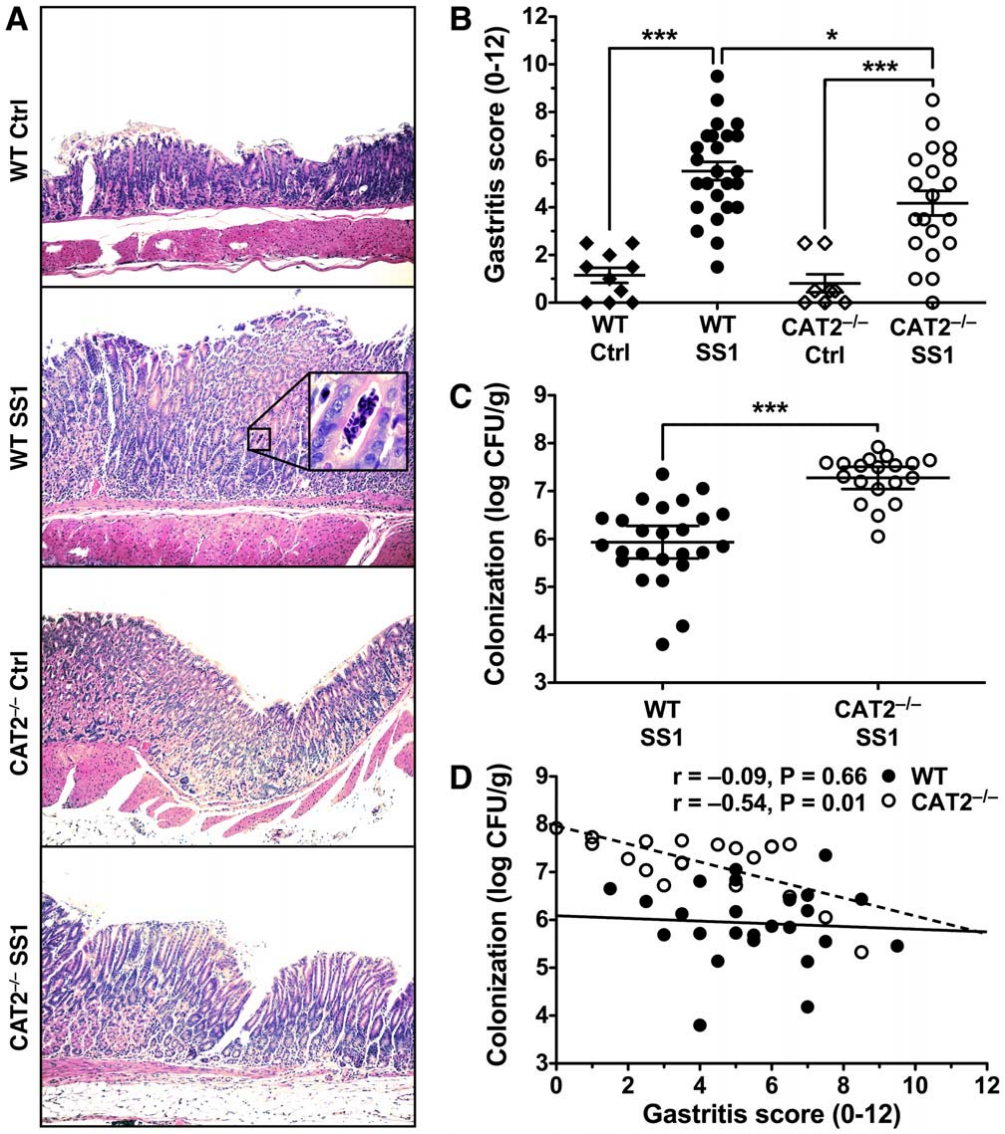
*H. pylori*-infected CAT2^−/−^ mice display less gastritis and increased gastric colonization. Wild type (WT) and CAT2^−/−^ mice were infected with *H. pylori* SS1 and sacrificed after 4 months. (A) Strips of stomach tissue containing both the body and the antrum were sectioned, mounted, and stained with hematoxylin and eosin. Representative sections of the antrum/body transition zone for each treatment group. Images were captured at 100× with the inset demonstrating a gland infiltrated with neutrophils (crypt abscess) at 600×. (B) Inflammation was scored on a 0–12 scale. Each point represents a single mouse (*n* = 8–25 per group). (C) In each *H. pylori*-infected mouse colonization levels were determined by quantification of colonies cultured from a piece of gastric body that was homogenized, diluted, and plated on selective medium. Each point represents a single mouse (*n* = 19–25 per group). (D) Colonization was plotted against gastritis score for each infected mouse. The lines illustrate the best-fit linear regressions obtained for the two strains of mice with the correlation coefficient and significance as indicated. In panels B and C, *, *p*<0.05; ***, *p*<0.001 for comparisons indicated.

We infected wild type and CAT2^−/−^ mice with *H. pylori* SS1 for 4 months and then inspected their stomach tissue. Histological examination revealed that infection caused gastritis in both strains of mice (Figure 1A, 1B). Importantly, there was a significant difference between the two strains of infected mice; the mean gastritis score dropped from 5.52±0.38 in wild type mice to 4.18±0.52 in CAT2^−/−^ mice. We also quantified *H. pylori* colonization levels in the infected mice. Average bacterial levels in wild type mice were 2.88×10^6^ CFU/g and this was significantly increased to 2.83×10^7^ CFU/g in CAT2^−/−^ mice (Figure 1C). In wild type mice, there was no correlation between colonization and gastritis when we plotted these variables (Spearman *r* = −0.09, *p* = 0.66); however, in CAT2^−/−^ mice increased colonization significantly correlated with decreased gastritis (*r* = −0.54, *p* = 0.01, Figure 1D). These findings indicate that CAT2 has a host defense role in the immune response to *H. pylori* infection, though it is insufficient to completely clear the bacteria.

### Acutely infected CAT2^−/−^ mice exhibit lower abundance of gastric macrophages and fewer activated dendritic cells

**Figure 2:**
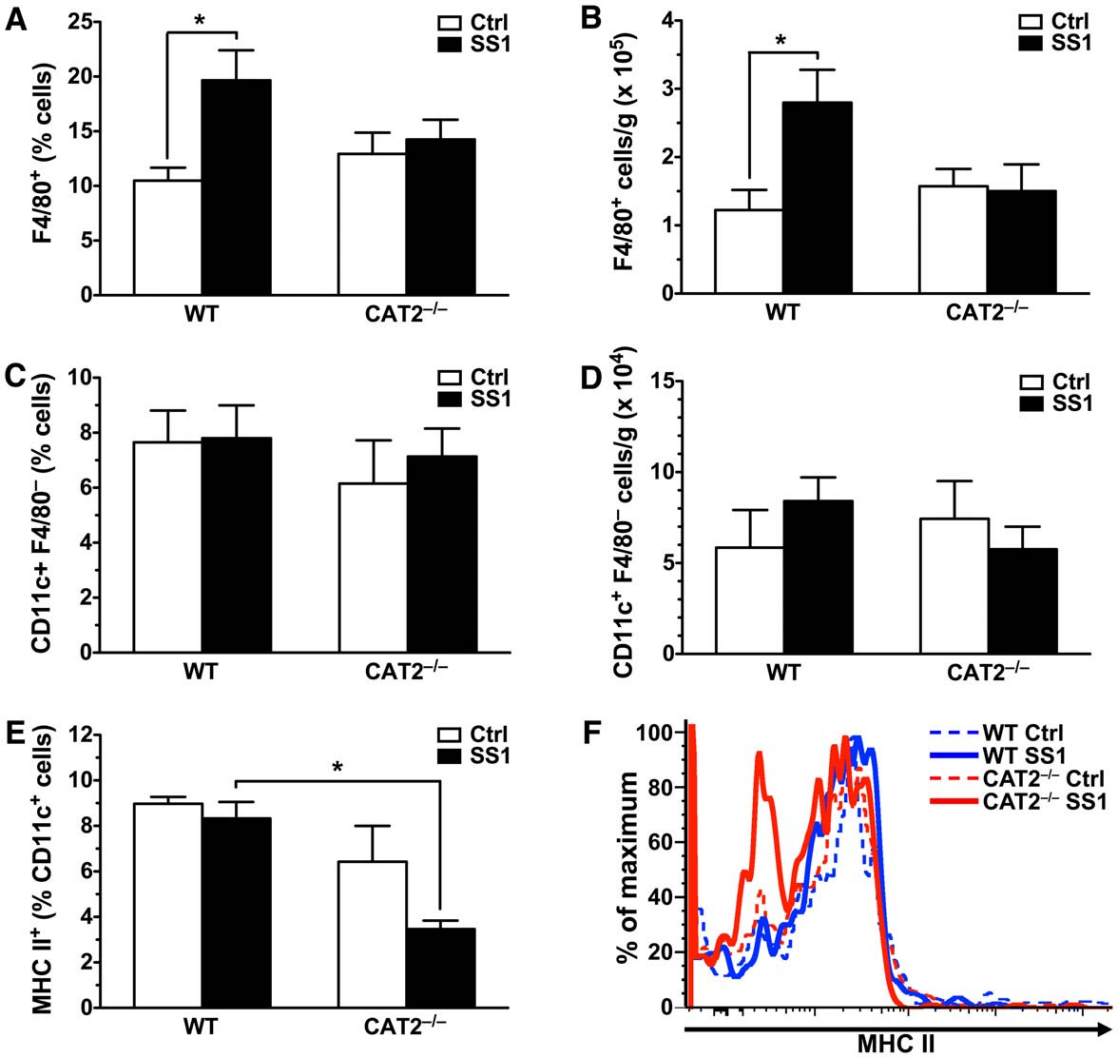
*H. pylori*-infected CAT2^−/−^ mice exhibit lower macrophage abundance and less dendritic cell activation. Gastric cells were isolated from mice infected for 48 h with *H. pylori* SS1 and were analyzed for expression of F4/80, a surface marker of macrophages, and CD11c, a marker of dendritic cells. (A) Percentage of F4/80^+^ cells per stomach (*n* = 8 per group). *, *p*<0.05 for the comparison indicated. (B) Absolute number of F4/80^+^ cells/g in these mice. (C) Percentage of CD11c^+^ F4/80^−^ cells per stomach (*n* = 4 per group). (D) Absolute number of CD11c^+^ F4/80^−^ cells/g in these mice. (E) CD11c^+^ cells were analyzed for surface expression of MHC II, a marker of activation of dendritic cells (*n* = 4 per group). *, *p*<0.05 for the comparison indicated. (F) Representative histograms of flow cytometric analysis indicating MHC II expression levels within the CD11c^+^ cells.

We next sought to determine the effects of CAT2 deletion on the innate immune response to *H. pylori* infection. We measured F4/80^+^ gastric macrophage levels in wild type and CAT2^−/−^ mice infected with *H. pylori* SS1 for 48 h, which our previous research has shown is the time point of peak macrophage levels [23], [24]. Analysis by flow cytometry indicated that while there was a significant increase in the percentage and absolute number of macrophages in wild type mice following acute *H. pylori* infection, no change was seen in macrophage levels in CAT2^−/−^ mice (Figure 2A and 2B, and Figure S1). We also measured CD11c^+^ F4/80^−^ dendritic cell levels in the stomachs of acutely infected mice. While we found no differences in the percentage or absolute number of dendritic cells regardless of the genotype of the mice or the infection status at this time point (Figure 2C and 2D, and Figure S1), there was a significant decrease in the percentage of MHC II^+^ dendritic cells in infected CAT2^−/−^ mice compared with their wild type counterparts (Figure 2E). This reduction was apparent in the representative histograms shown in Figure 2F as a leftward shift. This finding is noteworthy because surface expression of MHC II is a marker of activation and is indicative of dendritic cell participation in antigen presentation [25].

### Gastric macrophages in chronically infected CAT2^−/−^ mice express lower levels of iNOS protein

**Figure 3:**
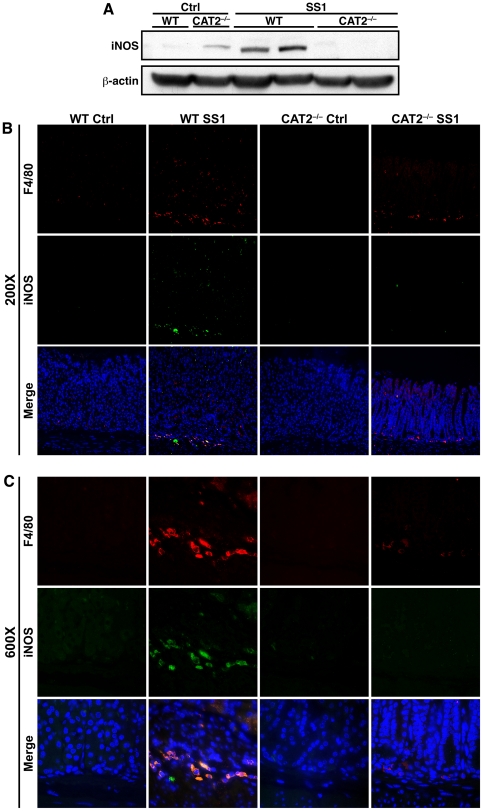
Chronically infected CAT2^−/−^ mice express lower levels of iNOS protein in gastric macrophages. (A) Protein was isolated from gastric tissue of mice infected for 4 months with *H. pylori* SS1 and from uninfected controls. Western blot analysis was performed to detect iNOS and β-actin in total tissue lysates. (B) Pieces of glandular stomach tissue from mice 4 months post-inoculation and controls were sectioned, mounted, and stained for immunofluorescence. F4/80 was detected with TRITC-tagged antibody (red), iNOS was identified with FITC-tagged antibody (green), and nuclei were stained with DAPI (blue). In the merged images yellow coloring surrounding blue nuclei indicates colocalization of F4/80 and iNOS within cells. Representative photomicrographs were captured at 200×. (C) High power images (600×) of the same sections.

We also examined changes to macrophages and iNOS after 4 months of infection. We isolated protein from gastric tissue lysates and performed Western blotting to detect iNOS. While iNOS protein expression was induced in infected wild type mice, levels were substantially decreased in infected CAT2^−/−^ mice (Figure 3A). We next examined iNOS expression in F4/80^+^ macrophages by immunofluorescent staining of tissue sections from chronically infected mice. *H. pylori* infection led to increased numbers of F4/80^+^ cells in both strains of mice, but the increase was greater in wild type than CAT2^−/−^ mice (Figures 3B and 3C, top row). Infected wild type mice also demonstrated more expression of iNOS protein in gastric tissue than CAT2^−/−^ mice (Figures 3B and 3C, middle row). When we merged the captured images, the majority of the iNOS expression was colocalized with F4/80^+^ cells in the wild type mice, and the CAT2^−/−^ mice exhibited an absence of iNOS^+^ macrophages (Figures 3B and 3C, bottom row). These data are an *in vivo* confirmation of our previous *in vitro* findings that L-Arg transport is essential for iNOS protein expression in *H. pylori*-stimulated macrophages [4]. We also stained tissue sections to colocalize iNOS expression in CD11c^+^ dendritic cells. Chronic infection increased dendritic cell numbers in both strains of mice (Figures S2A and S2B, top row). We again observed some iNOS-expressing cells (Figures S2A and S2B, middle row), but merging the images revealed no apparent overlap in the fluorescent signals (Figures S2A and S2B, bottom row), suggesting that dendritic cells are not a source of iNOS expression during *H. pylori* infection.

### IL-12p70 expression in response to infection is altered in CAT2^−/−^ mice

**Figure 4:**
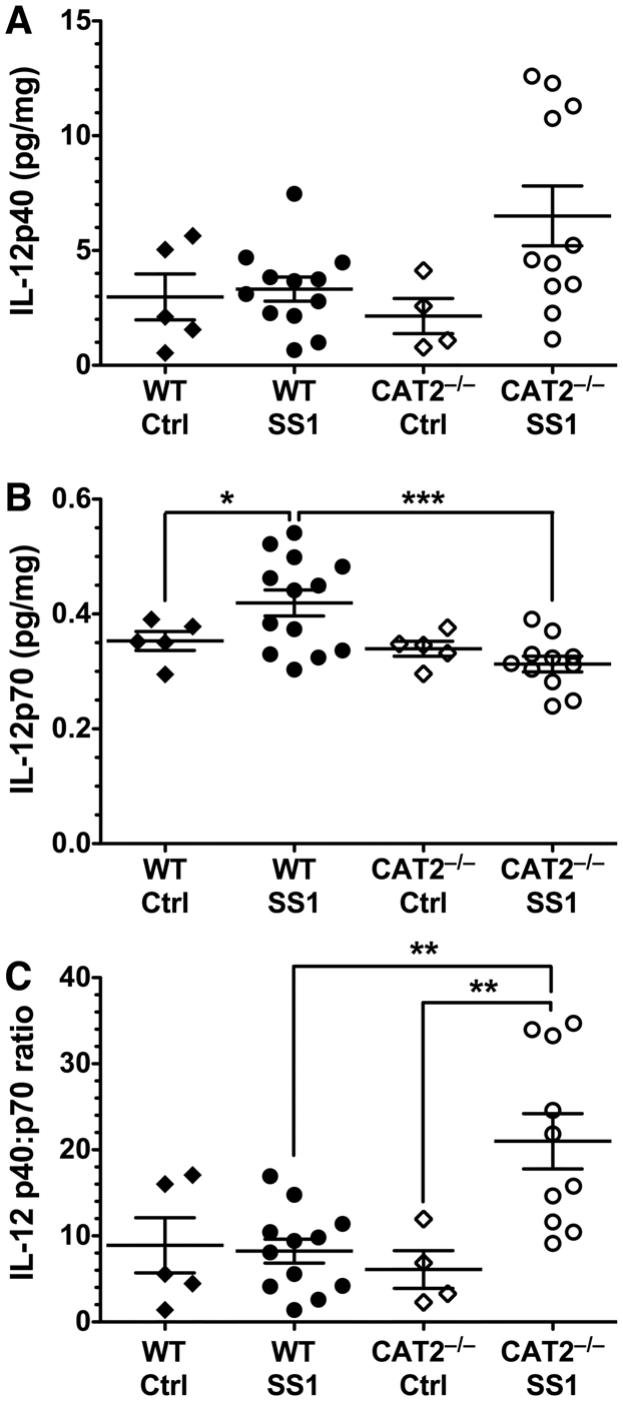
Expression of IL-12, a Th1-response regulator, in response to *H. pylori* infection is altered in CAT2^−/−^ mice. Protein lysates were obtained from gastric tissues from mice infected for 4 months with *H. pylori* SS1 or uninfected controls. Levels of IL-12 cytokines (normalized to total protein levels) were quantified by Luminex. (A) IL-12p40 levels. (B) IL-12p70 levels. (C) Ratios of IL-12p40 to IL-12p70. Each point represents a single mouse (*n* = 4–12 per group). *, *p*<0.05; **, *p*<0.01; ***, *p*<0.001 for comparisons indicated.

We next investigated if there were any alterations in IL-12 protein levels in gastric tissues that could be linked to the decreased immune response and control of bacterial levels observed in infected CAT2^−/−^ mice. We utilized Luminex technology to simultaneously measure multiple cytokines. Quantification of IL-12p40 revealed no differences in expression with infection in wild type mice, and a modest increase in CAT2^−/−^ mice (Figure 4A). When we measured levels of IL-12p70, the active heterodimer, we observed a significant increase following infection of wild type mice (Figure 4B), which was completely abrogated in infected CAT2^−/−^ mice. We also calculated the p40∶p70 ratio, as higher ratios have been associated with increased Th2 and decreased Th1 responses [26], [27]. Interestingly, we found no difference in this ratio between control and infected wild type mice (Figure 4C). However, infected CAT2^−/−^ mice exhibited a 3.45-fold increase in p40∶p70 ratio compared to uninfected CAT2^−/−^ mice, which was also 2.55-fold greater than levels seen in infected wild type mice. These findings suggest that CAT2 expression is linked to production of IL-12p70, which may enhance the Th1 response necessary for bacterial clearance.

### Cytokine expression in response to infection is altered in CAT2^−/−^ mice

**Figure 5:**
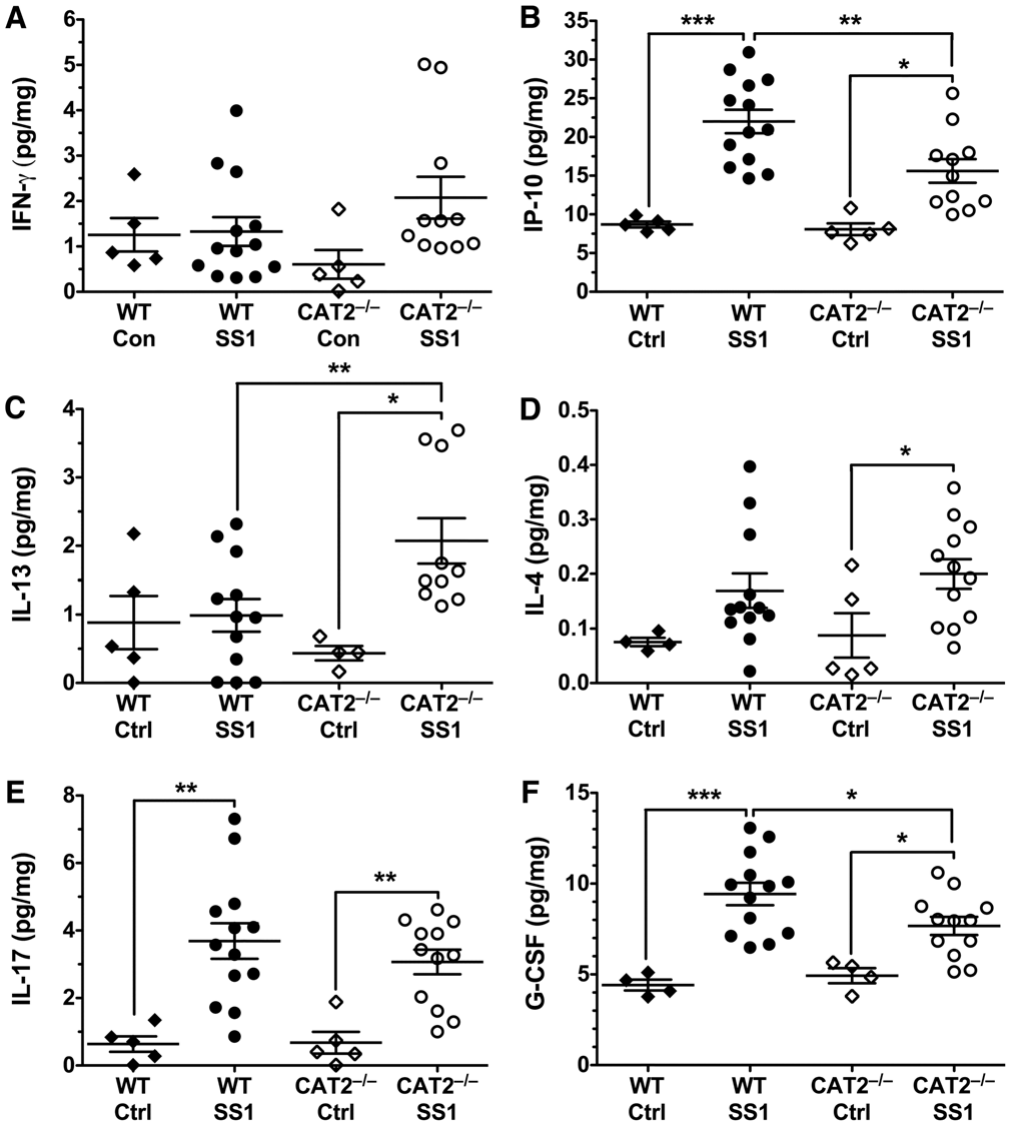
Th2, Th1, and innate response cytokine expression in response to *H. pylori* infection is altered in CAT2^−/−^ mice. Protein lysates were analyzed by Luminex for expression levels of other cytokines. (A) IFN-γ levels. (B) IP-10 levels. (C) IL-13 levels. (D) IL-4 levels. (E) IL-17 levels. (F) G-CSF levels. Each point represents a single mouse (*n* = 4–12 per group). *, *p*<0.05; **, *p*<0.01; ***, *p*<0.001 for comparisons indicated.

We also measured other cytokines in chronically infected gastric tissue from wild type and CAT2^−/−^ mice to identify divergences in response that could contribute to the differences we noted in gastritis and level of colonization. While quantification of IFN-γ revealed no differences between infected wild type and CAT2^−/−^ mice (Figure 5A), we found that levels of IFN-γ-induced protein 10 (IP-10), a Th1-associated chemokine, were altered. Expression levels were increased by 2.5–fold following infection in wild type mice, and were reduced by 48% in infected CAT2^−/−^ mice (Figure 5B). Th2 responses have classically been defined by their involvement in development of the humoral response to infection and ability to downregulate the Th1 response. IL-13, an important Th2 cytokine, has been implicated in the immune response to *H. pylori* [28]. In our experiments, infection of wild type mice had no effect on expression of IL-13 protein, while CAT2^−/−^ mice exhibited a significant, 4.3–fold increase following infection (Figure 5C). We also noted a significant increase in the Th2 cytokine IL-4 following infection of CAT2^−/−^ mice that was not observed in wild type mice (Figure 5D). Consistent with other reports [23], levels of the prototype Th17 cytokine IL-17 levels were increased in infected wild-type mice, but there were no differences between the infected wild-type and CAT2^−/−^ mice (Figure 5E). Similarly, the chemokines RANTES (CCL5) and KC (CXCL1) were increased with infection to a similar degree in wild type and CAT2^−/−^ mice (Figures S3A and S3B). As a whole, these data suggest that CAT2 deletion primarily has the effect of shifting the balance between Th1 and Th2 cytokines.

In addition to the macrophage and lymphocyte response, the active gastritis seen during *H. pylori* infection is significantly composed of granulocytes. As gastritis was lessened in CAT2^−/−^ mice we quantified levels of granulocyte-colony stimulating factor (G-CSF), which is involved in driving the proliferation of granulocytes, to determine if differences in its expression could be involved. After chronic infection, G-CSF levels in wild type mice increased by 2.1–fold (Figure 5F). The levels were significantly lower in infected CAT2^−/−^ mice. The observed alterations in cytokine expression in CAT2^−/−^ mice suggest that the transporter may act as a regulator of both the innate and adaptive arms of the immune response.

## Discussion

As a consequence of the inadequate immune response mounted during *H. pylori* infection, carriage is lifelong without antimicrobial treatment. Our previous work indicates that understanding the interplay of various pathways of arginine metabolism in the host will be critical to uncover the causes for this failure to clear the bacteria. We now show that the ability of the host to promote an innate and adaptive immune response, albeit one that is not completely effective, is enhanced by CAT2. We speculate that in the setting of CAT2 deletion, a feedback loop may exist in which the ability of *H. pylori* to interfere with the host's immune response allows increased bacterial proliferation that further enhances immune suppression.

We have reported that the bacterial induction of host Arg2 promotes immune evasion by the bacteria by limiting iNOS production of bactericidal NO and promoting macrophage apoptosis [29]. In infected Arg2^−/−^ mice we observed increased gastritis and reduced bacterial load, indicating a deleterious effect of this enzyme during the host's response to *H. pylori* infection [6]. In the current study we have demonstrated that CAT2, which is responsible for L-Arg transport during infection and is thus an upstream regulator of iNOS and Arg2, is also important in determining the host response during infection. Compared to wild type mice, infected CAT2^−/−^ mice exhibited increased levels of colonization and lower gastritis scores, suggesting that CAT2 has a stimulatory role in the immune response to *H. pylori*. This may also indicate that transport of L-Arg by this protein is essential for the immune response normally seen during infection and that augmentation of L-Arg uptake could be a useful strategy for therapeutic immunomodulation.

An inverse correlation between the extent of gastric inflammation and the level of *H. pylori* colonization has been reported, but only under conditions involving manipulation of immunity toward a more vigorous Th1 or Th17 response [30], [31], [32]. When we plotted these data from wild type mice in the present study we saw no relationship, but strikingly, we did observe a significant inverse correlation in infected CAT2^−/−^ mice. We found a similar result in our recent study using Arg2^−/−^ mice, no association in wild type mice and a negative correlation in the knockouts, which indicated that the defective immune response found normally was partially overcome when Arg2 was deleted [6]. One difference between these two experimental systems is that in Arg2^−/−^ mice the inverse correlation appears to be driven by the reduction in colonization in mice with high levels of gastritis [6], whereas in CAT2^−/−^ mice the effect is derived from the high levels of colonization in the setting of reduced gastritis. Thus, our data here suggest that the strongest effect of CAT2 is its capacity to control bacterial colonization.

In our earlier study we reported that infection led to an increase in the number of macrophages in wild type mice that was further increased in Arg2^−/−^ knockout mice [6]. We cited this as evidence of immune suppression attributable to Arg2 induction by *H. pylori*. In the current study, the increase in gastric macrophages in wild type mice was absent in CAT2^−/−^ mice, indicating that CAT2 may act in a pro-inflammatory manner in support of the immune response. Moreover, we noted that the increase in iNOS protein expression in chronically infected mice that was detected in wild type mouse tissues was absent in CAT2^−/−^ mice. These data are consistent with our previous findings in RAW 264.7 macrophages that inhibition of L-Arg transport with l-lysine, a competitive inhibitor of L-Arg uptake, or siRNA knockdown of CAT2 prevents *H. pylori*-stimulated induction of iNOS and killing of *H. pylori* [33]. By immunofluorescence, we localized iNOS expression within gastric macrophages during chronic *H. pylori* infection, and both macrophage cell numbers and iNOS levels were dramatically reduced in CAT2^−/−^ mice. These data suggest that increasing L-Arg transport in macrophages allows for more robust production of NO and a subsequent reduction in bacterial load. However, we have reported that the polyamine spermine inhibits iNOS protein translation [22] by inhibiting L-Arg uptake [4]. ODC, the rate-limiting enzyme for polyamine synthesis, is also upregulated in macrophages during *H. pylori* infection [4], [24]. Thus, we have found that supplementation of mice with L-Arg leads to increased polyamine levels in the infected mucosa that prevents enhancement of iNOS expression and therefore has no effect on colonization (data not shown).

Dendritic cells, monocytic cells that are activated by bacterial components and specialized for antigen presentation, are also involved in the immune response to *H. pylori*. Kao et al. reported that CD11c^+^ dendritic cells are found in the mouse stomach as early as 6 h post infection [9]. *H. pylori* induces dendritic cell activation and maturation, which includes upregulation of MHC II [10]. *H. pylori*-stimulated dendritic cells also reportedly express increased levels of inflammatory cytokines, especially IL-12p70, that can induce Th1 lymphocyte responses [9], [10], [34]. We found that infected CAT2^−/−^ mice exhibited a reduction in MHC II expression on dendritic cells, which could impair their ability to participate in the immune response, indicating that in wild type mice CAT2 may contribute to the dendritic cell response to *H. pylori*.

*H. pylori* induces a robust gastric inflammation that is nonetheless insufficient to eliminate the infection. Studies have indicated that T cells are required for development of gastritis, as inflammation does not arise in mice lacking these cells [31]. Specifically, a Th1 response is associated with increased inflammation and recruitment of monocytes that can eliminate the bacteria [3], [14]. The active form of IL-12, the p70 heterodimer, is composed of disulfide-linked p35 and p40 subunits, and promotes Th1 responses while simultaneously suppressing Th2 activation [26]. p40 is a pleiotropic molecule. When joined with p19 it forms the cytokine IL-23, which is involved in development of a Th17 response [35]. In addition, p40 monomers and homodimers are believed to act as binding antagonists for IL-12p70 [12], [36]. It has been reported that IL-12-deficent mice are unable to block *H. pylori* infection following vaccination [14], indicating that this cytokine is essential for a strong bacteria-eliminating immune response. In this study, we observed a significant increase in IL-12p70 following infection of wild type mice that was not observed in CAT2^−/−^ mice. A similar pattern of expression was noted for IP-10, which has been implicated as a chemoattractant for Th1 lymphocytes during *H. pylori* infection [37], while opposite effects on expression of the prototypical Th2 cytokine IL-13 were observed. We also found an increased p40∶p70 ratio in CAT2^−/−^ mice following infection. It has been reported that the immune suppression seen in older humans is accompanied by an increased p40∶p70 ratio [26]. In conjunction with our observation that CAT2^−/−^ mice exhibit significantly less gastritis than wild type mice, these findings suggest that L-Arg transport in macrophages is essential for development of the Th1 response during *H. pylori* infection.

*H. pylori* infection is characterized by a chronic acute gastritis that includes a constant presence of neutrophils. It has been reported that neutrophils are able to engulf and destroy bacteria *in vivo* [38], however this activity is normally insufficient to clear the infection. In a model system infecting IL-10^−/−^ mice with *H. felis*, depletion of neutrophils suppressed the Th1 immune response to infection [39], which suggests that granulocytes may also be involved in signaling the adaptive immune response. In the current study we found that following infection, compared with wild type mice, CAT2^−/−^ mice exhibited decreased expression of G-CSF, a growth factor that enhances neutrophil proliferation. Since we observed a concomitant reduction in inflammation in the CAT2^−/−^ mice, this finding may indicate that L-Arg transport via CAT2 plays a role in neutrophil recruitment to the site of infection.

Other reports have also indicated a role for CAT2 in the modulation of host immunity. Thompson et al. reported that CAT2^−/−^ mice infected with the parasite *Toxoplasma gondii* were more susceptible to disease than wild type mice and exhibited a decrease in NO production [40]. As observed during *H. pylori* infection, immunity to *T. gondii* also involves a Th1 response and is NO-dependent [41]. However, in contrast to our findings, cells isolated from *T. gondii*-infected CAT2^−/−^ mice exhibited increased expression of IFN-γ protein following ex vivo stimulation. Thompson et al. also studied the involvement of CAT2 in the immune response to the Th2-stimulating helminth *Schistosoma mansoni* and observed a similar increase in susceptibility to infection in CAT2^−/−^ mice [40]. These findings suggest that CAT2 plays important roles in multiple pathways of adaptive immunity. Apart from its importance in the response to infection, spontaneous inflammation and increased dendritic cell activation has been reported in the lungs of CAT2^−/−^ mice [25]. Thus, the role of CAT2 during inflammation appears to be specific to different tissues and disease models, and furthermore, investigations in models of inflammation-associated carcinogenesis could also yield important insights.

## Supporting Information

Figure S1 **Relative abundance of immune cells in mice acutely infected with** ***H. pylori*** **.** Gastric cells isolated from mice infected for 48 h with *H. pylori* SS1 were analyzed by flow cytometry for expression of F4/80 and CD11c. Representative dot plots indicate percentages of F4/80^−^ CD11c^+^ cells (upper left quadrant), F4/80^+^ CD11c^+^ cells (upper right quadrant), and F4/80^+^ CD11c^−^ cells (lower right quadrant) in wild type and CAT2^−/−^ mice.
(TIF)

Figure S2 **Lack of iNOS expression in dendritic cells in chronic** ***H. pylori*** **infection.** (A) Pieces of glandular stomach tissue from mice 4 months post-inoculation and controls were sectioned, mounted, and stained for immunofluorescence. CD11c was detected with phycoerythrin-tagged antibody (red), iNOS was identified with FITC-tagged antibody (green), and nuclei were stained with DAPI (blue). There is no yellow cytoplasmic coloring in the merged images indicating an absence of colocalizing CD11c and iNOS within cells. Representative photomicrographs were captured at 200×. (B) High power images (600×) of the same sections.
(TIF)

Figure S3 **Expression of chemokines induced by** ***H. pylori*** **infection is insensitive to the presence of CAT2.** Protein lysates were analyzed by Luminex for expression levels of other cytokines. (A) RANTES levels. (B) KC levels. Each point represents a single mouse (*n* = 4–13 per group). **, *p*<0.01; ***, *p*<0.001 for comparisons indicated.
(TIF)
