# Supplementary figures and images for: Cationic Amino Acid Transporter 2 Enhances Innate Immunity during Helicobacter pylori Infection
Source: PLoS One. 2011 Dec 14;6(12):e29046. doi: 10.1371/journal.pone.0029046 (PMC3237590; doi:10.1371/journal.pone.0029046)

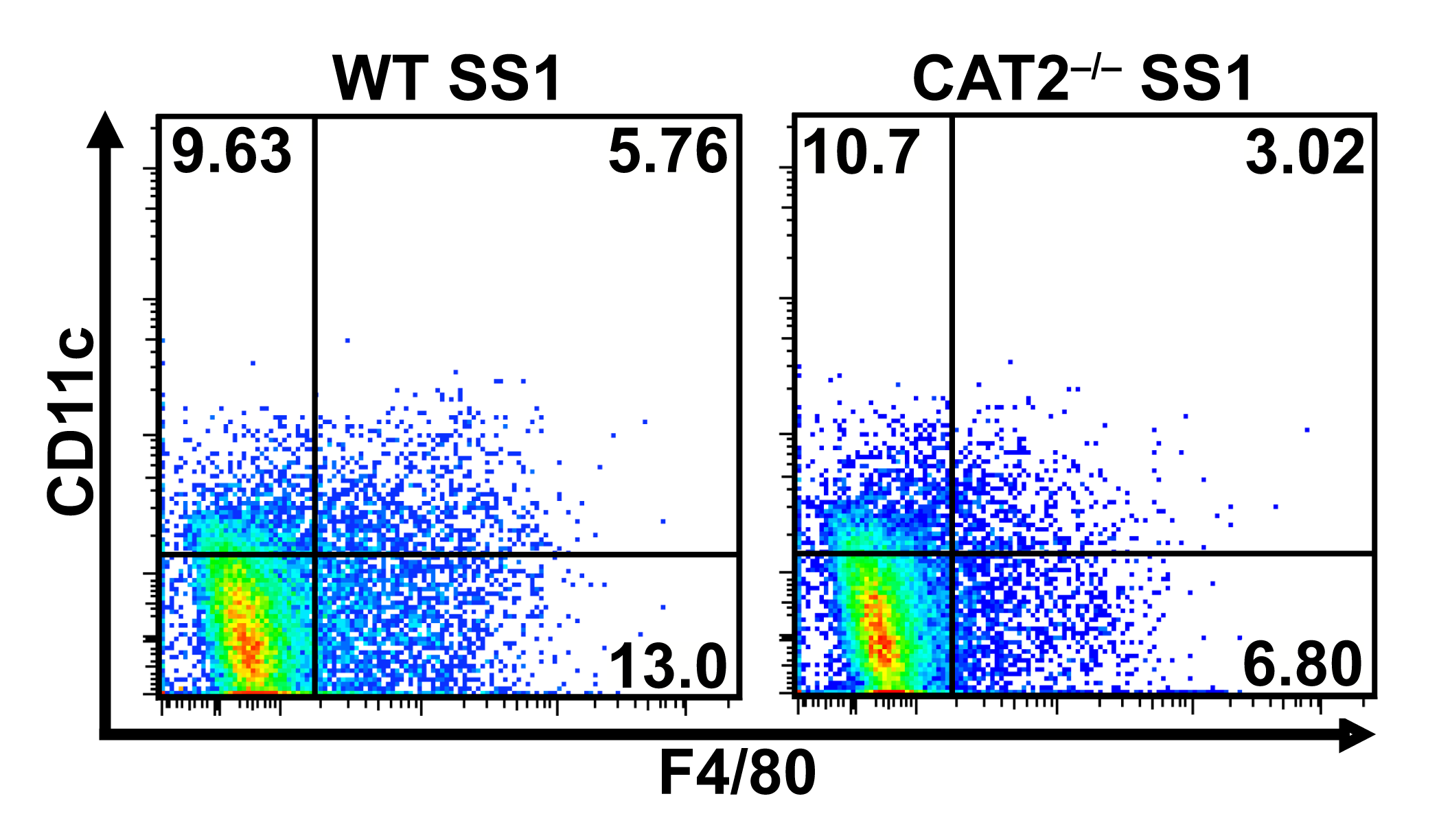

Supplement: Figure S1 — Relative abundance of immune cells in mice acutely infected with H. pylori . Gastric cells isolated from mice infected for 48 h with H. pylori SS1 were analyzed by flow cytometry for expression of F4/80 and CD11c. Representative dot plots indicate percentages of F4/80− CD11c+ cells (upper left quadrant), F4/80+ CD11c+ cells (upper right quadrant), and F4/80+ CD11c− cells (lower right quadrant) in wild type and CAT2−/− mice. (TIF) [file pone.0029046.s001.tif]

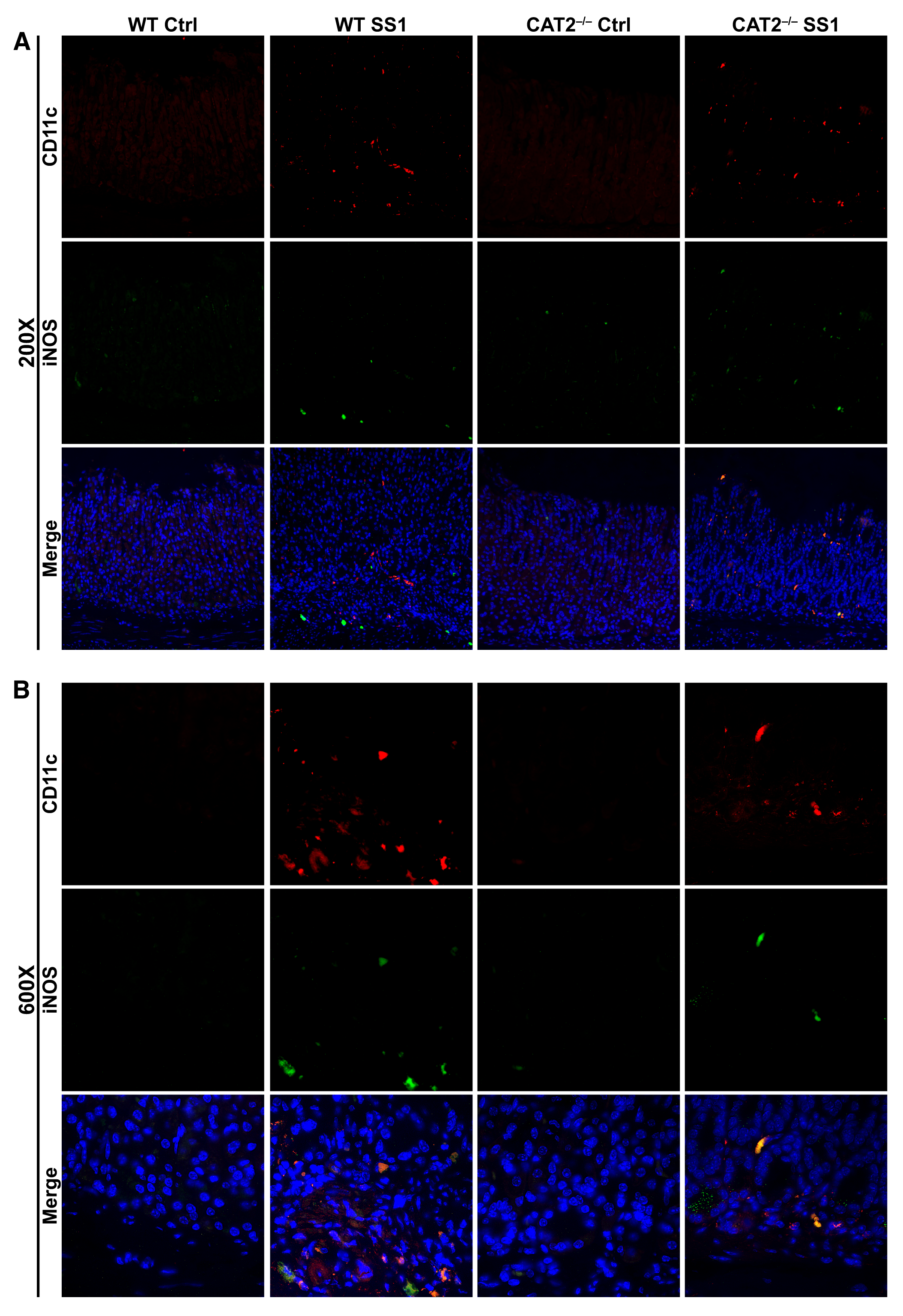

Supplement: Figure S2 — Lack of iNOS expression in dendritic cells in chronic H. pylori infection. (A) Pieces of glandular stomach tissue from mice 4 months post-inoculation and controls were sectioned, mounted, and stained for immunofluorescence. CD11c was detected with phycoerythrin-tagged antibody (red), iNOS was identified with FITC-tagged antibody (green), and nuclei were stained with DAPI (blue). There is no yellow cytoplasmic coloring in the merged images indicating an absence of colocalizing CD11c and iNOS within cells. Representative photomicrographs were captured at 200×. (B) High power images (600×) of the same sections. (TIF) [file pone.0029046.s002.tif]

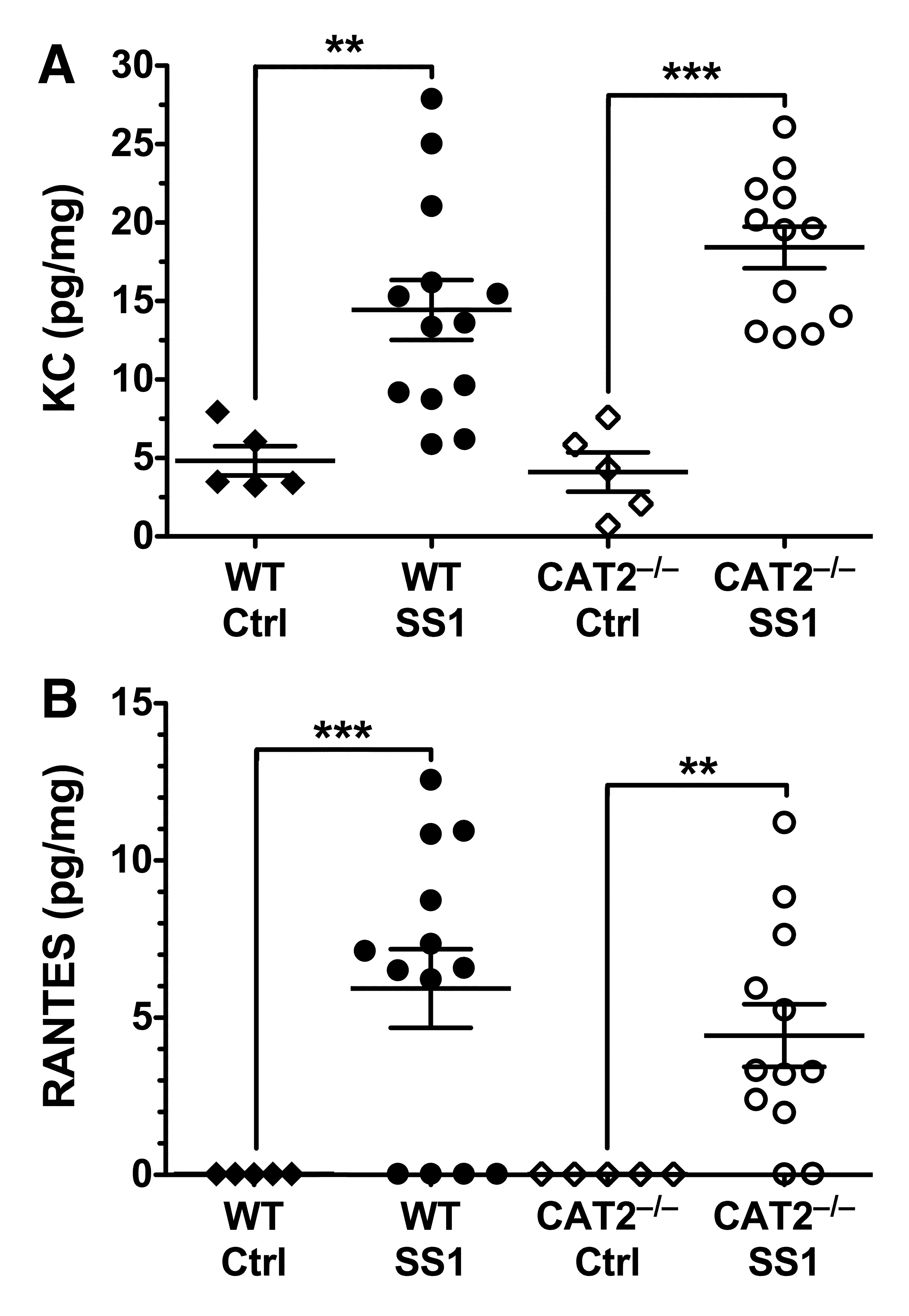

Supplement: Figure S3 — Expression of chemokines induced by H. pylori infection is insensitive to the presence of CAT2. Protein lysates were analyzed by Luminex for expression levels of other cytokines. (A) RANTES levels. (B) KC levels. Each point represents a single mouse (n = 4–13 per group). **, p<0.01; ***, p<0.001 for comparisons indicated. (TIF) [file pone.0029046.s003.tif]
